# Thyroidectomy Complicated by Severe Septic Shock Due to *Streptococcus pyogenes*: A Case Study

**DOI:** 10.3390/jcm14196742

**Published:** 2025-09-24

**Authors:** Edyta Zagrodnik, Sylwia Wieder-Huszla, Anna Południewska, Marta Górecka, Anna Surówka, Patrycja Krężel, Anna Jurczak

**Affiliations:** 1 Clinical Department of Anesthesiology and Intensive Care for Adults and Children, Pomeranian Medical University in Szczecin, University Clinical Hospital No. 1, 71-252 Szczecin, Poland; edyta.zagrodnik@pum.edu.pl (E.Z.); marta.gorecka@pum.edu.pl (M.G.); 2 Department of Specialized Nursing, Pomeranian Medical University in Szczecin, 71-210 Szczecin, Poland; anna.poludniewska@pum.edu.pl (A.P.); anna.jurczak@pum.edu.pl (A.J.); 3 Department of Plastic, Endocrine, and General Surgery, University Clinical Hospital No. 1, 72-010 Police, Poland; anna.surowka@pum.edu.pl

**Keywords:** septic shock, cardiac arrest, *Streptococcus pyogenes*, strumectomy

## Abstract

**Background**: In recent years, the incidence of thyroid cancer has been increasing due to environmental, biological, and genetic factors. One of the treatment methods for thyroid cancer is strumectomy, a procedure associated with a high risk of postoperative complications. **Methods**: The following case presents septic shock with sudden cardiac arrest and cardiopulmonary resuscitation, caused by *Streptococcus pyogenes*, in a 45-year-old woman who underwent surgery for papillary carcinoma. **Results**: The patient required long-term interdisciplinary treatment in the Clinical Department of Anesthesiology and Intensive Care. **Conclusions**: Necrotizing mediastinitis after thyroidectomy caused by *Streptococcus pyogenes* is a rare and dangerous complication. The analysis of the case proves how important the preoperative assessment of the patient’s health (especially taking a detailed history) and the patients’ awareness of possible risk factors are. An equally important aspect is the awareness of the therapeutic team regarding the transmission of infections, their dynamics, and the consequences of the procedures undertaken.

## 1. Introduction

Thyroid cancer cases have been on the rise in recent years. In 2020, according to Global Cancer Statistics (GLOBOCAN) data, more than 580,000 new cases of this cancer were diagnosed worldwide. In Poland, there is a steady increase in thyroid cancer, with about five thousand new cases diagnosed annually. The peak incidence is in the 4–5th decade of life and more often affects women [1,2]. The cause of the increase in the incidence of the disease is complex. Environmental, biological, and genetic factors play a major role. One of the decisive environmental factors was the discontinuation of iodine prophylaxis in 1980, which contributed to the increase in the incidence, especially of follicular cancer. In turn, a large number of cases of papillary thyroid cancer were caused by the nuclear power plant failure in Chernobyl in 1986, which led to the contamination of Poland with radioactive iodine [3,4,5]. Biological and genetic factors include female sex, family history of thyroid cancer, the use of contraception, and hormone therapy [6].

There are several types of thyroid cancer. The vast majority, i.e., 80–90% of cases, are papillary and follicular carcinomas. The remaining 10% of cases are medullary and anaplastic cancers. In contrast, sarcomas, lymphomas, and metastases are relatively rare [7,8]. Thyroid cancers have a benign course and a good prognosis. With properly selected treatment, 10-year survival is possible in 80% of cases [9].

Thyroidectomy is a surgical method of treatment that is recommended for benign as well as malignant disease of the thyroid [10]. Surgical treatment remains the preferred option for treating various thyroid diseases [11]. Despite existing controversies regarding the choice of the best surgical treatment, both total thyroidectomy (TT) and subtotal thyroidectomy (STT) have remained the two main surgical procedures [12].

The indications for thyroidectomy are the suspicion or diagnosis of thyroid cancer and the presence of compression symptoms. The compression symptoms include respiratory disorders, fatigue, a feeling of pressure on the respiratory tract, cough, and dysphonia. Compression symptoms result from the pressure of the thyroid goiter on the respiratory tract and are a serious symptom that requires therapeutic measures [13].

Thyroidectomy is a procedure with a high risk of postoperative complications. These include intraoperative bleeding, damage to the recurrent laryngeal nerve, hypoparathyroidism, laryngeal edema, postoperative wound suppuration, and esophageal perforation [14,15,16,17]. Septic shock, on the other hand, according to the 2016 definition, is a multi-organ dysfunction that poses a life-threatening risk to the patient due to an impaired regulation of the body’s response to infection [18].

The case presented here is a description of septic shock in a 45-year-old woman with papillary carcinoma diagnosed by histopathology after total thyroidectomy, complicated by *Streptococcus pyogenes* infection, requiring long-term interdisciplinary treatment in the intensive care unit.

## 2. Case Presentation

A 45-year-old patient was admitted to the Clinical Department of Anesthesiology and Intensive Care as an emergency from the Central Operating Theater after a revision surgery of the strumectomy bed with suspected mediastinal abscess. The patient had previously undergone elective total strumectomy due to suspected thyroid cancer. On the first postoperative day, she reported chest pain. She consulted with an internist―acute coronary syndrome was excluded. The following day, from the afternoon hours, there was a dynamic deterioration of the general condition, with tachycardia, hypotension, and desaturation despite passive oxygen therapy; clinical features of shock with respiratory and circulatory failure; biochemically increasing inflammatory parameters; and renal dysfunction.

Due to cyanosis of the face, neck, and chest, an urgent angio-CT scan of the chest was performed using the pulmonary embolism protocol, which excluded pulmonary embolism. It showed bilateral pleural effusion, suspected pneumonia, and mediastinitis. Noradrenaline infusion and empirical antibiotic therapy were initiated. After the procedure, the patient was not woken up and was transferred to the intensive care unit (ICU).

On admission to the ICU, the general condition was extremely severe and labile. Clinical and biochemical symptoms of multiple organ failure in the course of septic shock as a result of nasopharyngitis, the wound, pneumonia, and pleural empyema with the etiology of *Streptococcus pyogenes* confirmed by microbiological test results obtained after 24 h. In the ward, the patient was unconscious, under the influence of general anesthetics, and intubated. Circulation was inefficient, labile, and centralized, supported by infusion of catecholamines. Capillary refill time (CRT) was undetectable, with a cool circumference, and cyanotic extremities. In the laboratory, arterial blood gasometry revealed severe hyperlactamic acidosis, hyperphosphatemia, elevated inflammatory parameters, leukopenia with lymphopenia and neutrophilocytosis, mild anemia, progressive thrombocytopenia, coagulopathy, malnutrition, hypoalbuminemia and hypoproteinemia, elevated amylase, renal failure parameters, cardiac markers, rhabdomyolysis, decreased FT3 levels, and proteinuria. Bedside ultrasound examination of the pleural cavities revealed bilateral hydrothorax (right > left) and lung atelectasis due to compression. Ultrasound-guided thoracentesis was performed removing 1000 mL from the right pleural cavity and 700 mL from the left. The fluid was purulent-serous and turbid in appearance. Samples were collected for general analysis (empyema with leukocytosis and Gram-positive granulomas) and microbiological examination (Table 1).

Invasive monitoring of vital functions typical of ICU conditions was implemented. A dialysis catheter was inserted, and continuous renal replacement therapy (CRRT) was initiated in continuous veno-venous hemodiafiltration (CVVHDF) mode with regional citrate anticoagulation (Ci-Ca). A gastric tube was inserted, draining approximately 1000 mL of stagnant contents. Triple analgosedation was administered, along with catecholamine infusions (epinephrine, norepinephrine, and dobutamine). Until microbiological test results were available, empirical broad-spectrum antibiotic therapy was initiated (meropenem, vancomycin, clindamycin, and metronidazole). Steroids, crystalloid infusions, regular insulin, potassium chloride, and protective therapy were also administered. Measures for edema prevention included elevated head positioning. Neuroprotective therapy and albumin infusion were additionally provided. A positioning regimen was implemented.

Over the following five days, the patient remained in a critically severe condition. Despite intensive treatment, no stabilization or improvement in her general condition was achieved. Escalation of intravenous catecholamine infusion was required. The skin was entirely covered with papular eruptions accompanied by erythema and epidermal peeling consistent with streptococcal toxemia. Locally, on the chest, necrotic lesions with epidermolysis and bullae were observed. The skin and mucous membranes exhibited features typical of scarlet fever. Microbiological examination yielded a positive wound culture for *Streptococcus pyogenes*. Based on this result, antibiotic therapy was modified; vancomycin and metronidazole were discontinued, and ampicillin was added. A detailed history was obtained from the family, revealing that the patient had contact with a child suffering from scarlet fever six days prior to hospital admission. Plasma, packed red blood cells, and platelet concentrates were transfused. Phosphorus supplementation and correction of electrolyte imbalances were performed. Follow-up laboratory tests were ordered. Renal replacement therapy was continued using the CytoSorb extracorporeal blood purification system, resulting in a significant reduction in inflammatory parameters measured at six-hour intervals. Selected parameter measurements are summarized in Table 2.

The patient was consulted with the Hyperbaric Medicine Center in Gdańsk but was disqualified from transport due to hemodynamic instability. She underwent multiple surgical consultations for an open wound with necrotic foci following thyroid gland removal. A cardiology consultation was also performed. Bilateral pleural drainage was implemented.

On the sixth day, relative clinical and biochemical stabilization of the patient was achieved. Laboratory tests showed a gradual regression of inflammatory markers, with neutrophilia and lymphopenia; mild anemia; decreasing D-dimer levels; malnutrition; hypoalbuminemia; hypoproteinemia; elevated amylase and aminotransferase levels; rhabdomyolysis; increased cardiac biomarkers; and decreased thyroid hormone levels.

Ultrasound examination of the pleura and lungs revealed a recurrent bilateral hydrothorax (right > left) and compression-related lung atelectasis. Follow-up CT imaging of the head and chest showed no abnormalities in the brain. Fluid was present in both pleural cavities with atelectasis of the adjacent lung segments. Numerous dorsal pulmonary consolidations were observed, consistent with atelectatic-inflammatory changes. Additionally, scattered micronodular lesions were noted. Subtle infiltrates were seen in the anterior mediastinal fat along the manubrium of the sternum, without signs of progression. Significant regression of gas in the soft tissues of the surgical area was noted. The ascending aorta appeared dilated, and the right atrium was enlarged. Free fluid was present in the peritoneal cavity.

On the seventh day, following a telephone consultation with a thoracic surgeon, the left pleural drain was removed due to obstruction. Under ultrasound guidance, a new pleural drain was inserted in the posterior axillary line at the sixth intercostal space. The procedure was complicated by inadvertent placement of the drain into the peritoneal cavity, resulting in splenic injury. Due to ongoing bleeding, the patient qualified for emergency laparotomy. Splenectomy was performed, and the splenic pedicle was secured. Perioperatively, the patient received transfusions of six units of packed red blood cells (PRBC), three units of platelet concentrate, and six units of fresh frozen plasma (FFP).

In the following days, after sedation was reduced, catecholamines were discontinued, and clinical as well as biochemical status improved, the patient was extubated. However, due to impaired cough reflex and difficulties with expectoration, reintubation was required.

The consultations conducted:

- The patient was routinely consulted by a thoracic surgeon, who performed pleurodesis of the left pleural cavity.
- Consulted endocrinologically due to hypothyroidism.
- Consulted by a speech therapist to improve swallowing mechanisms, expectoration, articulation, and head stabilization.
- At the base of the wound, to the left of the trachea, an opening with leakage of mucous content was observed, raising suspicion of a tracheal fistula. Consultations were held with thoracic surgery, general surgery, and otolaryngology specialists. CT imaging of the neck and chest confirmed a tracheocutaneous fistula.
- Due to the thinned, bacterially overloaded skin of the neck with focal necrotic changes and risk of anterior neck wall collapse, the patient was consulted by a wound care and advanced dressing specialist. She qualified for unconventional therapy—larval debridement therapy—after prior skin preparation with prescribed dressings and agents. During larval therapy, the patient was reintubated. Initial wound debridement—wound necrectomy—was performed, and a dressing with 150 larvae was applied, resulting in a very good effect.

Upon completion of larval therapy, vacuum-assisted closure (VAC) negative pressure wound therapy was initiated, with air leakage observed from the respiratory tract through the tracheal fistula. Subsequently, mechanical and chemical debridement was performed, along with the application of silver ion-containing dressings. The patient was extubated and maintained on spontaneous respiration. Gradual wound healing and progressive closure of the tracheocutaneous fistula were observed.

In the following days, the patient was evaluated by a neurologist due to a sensation of weakness and numbness in the left lower limb, which intensified during walking. Bilateral lower limb muscle weakness was noted, with a predominant lack of plantar flexion and tremors (probably drug-induced). Critical illness polyneuropathy was suspected. Continued rehabilitation was recommended, along with electromyography (EMG) and magnetic resonance imaging (MRI), which revealed no significant abnormalities.

An elective surgical procedure was performed involving skin autografting over granulating wounds, with a free area left above the tracheal fistula.

The patient was repeatedly consulted by endocrinologists, and her supplementation therapy was adjusted due to postoperative hypothyroidism and hypoparathyroidism.

By the time of discharge from the ICU, the patient was conscious and hemodynamically and respiratorily stable and reported no pain. She was actively undergoing rehabilitation—walking independently and performing daily activities without assistance. After determining the destination department, the patient was transferred as planned to the Department of Plastic, Endocrine, and General Surgery for further treatment.

## 3. Discussion

The presented case is an example of total thyroidectomy due to thyroid cancer complicated by a very severe extra-hospital *Streptococcus pyogenes* infection in a patient who had contact with a child with scarlet fever six days before the surgery. The planned surgery was performed in an asymptomatic period, with normal laboratory parameters. In the interview, the patient did not provide any information about contact with a sick person in the pre-hospital period. The post-operative period was complicated by septic shock with multi-organ failure. Septic shock as a complication of strumectomy is an extremely rare complication [14,15,16,17]. *Streptococcus pyogenes* (Strep A) is a bacterial pathogen causing a wide range of diseases, namely superficial infections (skin, throat), invasive infections (sepsis, skeletal infections), and immune-mediated diseases [19]. This infection contributes to significant social costs, antibiotic use, and, if not treated early, serious consequences [20]. Abundant epidemiological evidence confirms the frequent transmission of *Streptococcus pyogenes* during close contacts in both households and communities. Therefore, understanding the dynamics of infection transmission should be an important element of consideration for interdisciplinary teams at every stage, from planning to assessing the impact of the interventions undertaken [21,22].

The most common complications after strumectomy include intraoperative bleeding, damage to the recurrent laryngeal nerve, hypoparathyroidism, laryngeal edema, and esophageal perforation [14,15,16,17]. Surgical site infection after this type of surgery is an uncommon complication, which is usually localized around the incision, because this surgery is considered a clean procedure. According to data from PubMed, infection after strumectomy can spread, causing necrotizing mediastinitis. Although these are extremely rare complications, they can end in death [23,24,25,26,27]. The mortality rate is high, as it settles at 50%. Descending necrotizing mediastinitis (DNM) spreads along the cervical fascia, which is anatomically divided into three components: the pretracheal, perivascular, and prevertebral spaces. Infection is favored by gravity, breathing, and negative intrathoracic pressure [26].

In the case described, the intensive interdisciplinary treatment was successful, despite the high mortality rate among those infected with *Streptococcus pyogenes* [28,29,30]. It should also be emphasized that despite eight minutes of cardiopulmonary resuscitation, the patient left the ICU without neurological deficits, conscious, in full logical contact, following orders, and without pain complaints. Also noteworthy was the patient’s use of unconventional larval therapy, which is still a developing method of treating hard-to-heal wounds in the outpatient setting and is not used in the hospital setting [31]. The successful choice of this treatment method in the case of necrotic tissue of the postoperative bed with a coexisting tracheocutaneous fistula indicates new treatment options within the inpatient setting. There is no case report in the available literature on the use of maggots in the treatment of tracheal fistula.

## 4. Conclusions

Necrotizing mediastinitis after thyroidectomy caused by *Streptococcus pyogenes* is a rare and dangerous complication. The analysis of the above case proves how important the preoperative assessment of the patient’s health (especially taking a detailed history) and the patients’ awareness of possible risk factors are. An equally important aspect is the awareness of the therapeutic team regarding the transmission of infections, their dynamics, and the consequences of the procedures undertaken.

## Figures and Tables

**Table 1:** Selected laboratory parameters of the patient during her stay in the ICU.

Parameter	Admission Day	Third Day	Sixth Day
Leukocytes	1.28 thousand/uL	19.52 thousand/uL	29.37 thousand/uL
Erythrocytes	3.71 million/uL	2.74 million/uL	3.33 million/uL
Hemoglobin	11.3 g/dL	8.3 g/dL	10.0 g/dL
Hematocrit	34.2%	25.0%	30.2%
Platelets	159 thousand/uL	35 thousand/uL	25 thousand/uL
Creatinine	2.97 mg/dL	1.60 mg/dL	1.12 mg/dL
eGFR	17.1 mL/min/1.73 m^2^	34.9 mL/min/1.73 m^2^	52.6 mL/min/1.73 m^2^
Urea	68.40 mg/dL	25.20 mg/dL	47.50 mg/dL
Inorganic phosphorus	3.71 mmol/L	0.57 mmol/L	0.67 mmol/L
Procalcitonin	32.660 ng/mL	95.86 ng/mL	8.36 ng/mL
CRP	324.03 mg/L	344.14 mg/L	177.68 mg/L
AST	11 U/L	201 U/L	537 U/L
ALT	30 U/L	145 U/L	885 U/L
Amylase	150 U/L	109 U/L	73 U/L
Phosphocreatine kinase	417 U/L	7533 U/L	1164 U/L
Total protein	3.96 g/dL	3.95 g/dL	4.22 g/dL
Albumin	1.93 g/dL	2.46 g/dL	3.28 g/dL
Total cholesterol	57.40 mg/dL	31.90 mg/dL	71 mg/dL
Triglycerides	79.00 mg/dL	94.50 mg/dL	112 mg/dL
APTT	103 s	92.40 s	52.70 s
INR	1.83	1.71	1.39
Fibrinogen	431 mg/dL	595 mg/dL	212 mg/dL
D-dimer	5.05 µg/mL	6.22 µg/mL	3.07 µg/mL

**Table 2:** Selected laboratory parameters of the patient using CytoSorb.

Parameter	Initial Value	Value During Treatment	Final Value
IL-6	672 pg/mL	359 pg/mL	166 pg/mL
Lactates	56.80 pg/mL	51.20 pg/mL	25.4 pg/mL
CRP	254 mg/L	191 mg/L	177 mg/L
Phosphocreatine kinase	3468 U/L	1835 U/L	1164 U/L
Leukocytes	19.93 thousand/uL	23.18 thousand/uL	29.37 thousand/uL
Procalcitonin	65.08 ng/mL	19.52 ng/mL	7.17 ng/mL
Fibrinogen	595 mg/dL	394 mg/dL	212 mg/dL

## Data Availability

The original contributions presented in this study are included in the article. Further inquiries can be directed to the corresponding authors.

## Abbreviations

The following abbreviations are used in this manuscript:

GLOBOCAN	Global Cancer Statistics
ICU	Intensive care unit
CRT	Capillary refill time
CRRT	Continuous renal replacement therapy
CVVHDF	Continuous veno-venous hemodiafiltration
Ci-Ca	Citrate anticoagulation
CT	Computed tomography
PRBC	Packed red blood cells
FFP	Fresh frozen plasma
VAC	Vacuum-assisted closure
EMG	Electromyography
MRI	Magnetic resonance imaging
Strep A	*Streptococcus pyogenes*
DNM	Descending necrotizing mediastinitis
